# The Extracts of Cinnamon and Clove as Potential Biofungicides against Strawberry Grey Mould

**DOI:** 10.3390/plants9050613

**Published:** 2020-05-11

**Authors:** Lina Šernaitė, Neringa Rasiukevičiūtė, Alma Valiuškaitė

**Affiliations:** Laboratory of Plant Protection, Institute of Horticulture, Lithuanian Research Centre for Agriculture and Forestry, LT-54333 Babtai, Kaunas district, Lithuania; neringa.rasiukeviciute@lammc.lt (N.R.); alma.valiuskaite@lammc.lt (A.V.)

**Keywords:** *Botrytis cinerea*, biocontrol, biofungicides, detached strawberry leaves, inhibition, plant extracts

## Abstract

Biofungicides from plants are a possibility for the biocontrol of fungal diseases, as chemical products may be harmful to the environment and humans. Strawberry is one of the many plants infected by grey mould (*Botrytis cinerea*), and innovative methods of biocontrol against *B. cinerea* are under investigation. Clove (*Syzygium aromaticum* L.) and cinnamon (*Cinnamomum cassia* L.) accumulate natural compounds, such as eugenol and cinnamaldehyde, which provide antimicrobial and antifungal properties; thus, extracts of these plants could be possibly used as biofungicides. During this study, the inhibition of *B. cinerea* by clove and cinnamon extracts was evaluated in vitro on Petri plates and detached strawberry leaves; additionally, the chemical composition of volatiles was identified. Clove extract consisted of 52.88% eugenol, and cinnamon consisted of 74.67% cinnamaldehyde. The efficacy of the extracts on detached strawberry leaves showed that 12 mL L^−1^ concentration of clove extract was effective in suppressing the grey mould infection. Clove and cinnamon extracts showed an equal ability to inhibit *B. cinerea* on Petri plates. However, the results of the detached strawberry leaves assay showed that clove extract was more effective as a biocontrol product. Overall, clove extract expressed a high potential for application in biofungicides formulations.

## 1. Introduction

Grey mould caused by *Botrytis cinerea* has a significant economic impact on plant production for a variety of different crops [1,2,3,4,5]. Strawberry is one of the hosts and can be damaged during different growth stages. Plant protection measures against fungal pathogens are necessary in farms in order to avoid yield and crop loss. However, chemical fungicides leave residues in the environment and in agricultural products and indirectly cause harm to humans. Following the guidance of integrated pest management, innovative ways to control pathogens should be found, with the aim of making agriculture more sustainable. Grey mould is conventionally controlled by chemical fungicides [6], and biocontrol products for strawberry are in demand [7].

Cinnamon (*Cinnamomum cassia* L.) extracts and essential oils have been investigated for their various medicinal properties [8,9,10,11,12]. For example, the cinnamon extract was examined for antifungal effects in bandages against human-pathogenic fungi [13]. Clove (*Syzygium aromaticum* L.) has been used in traditional medicine [14]. There are many reports on its antimicrobial and broad antifungal activities in the literature [2,15,16,17,18,19,20].

Plants accumulate secondary aromatic metabolites that have antimicrobial or antifungal activities [21]. As naturally occurring compounds may be an option for controlling fungal diseases [6,22,23], attention should be paid to the composition of natural compounds from plants and their extraction possibilities. The main constituent of clove essential oil is eugenol [24,25,26,27,28]. There are studies reporting the effectiveness of eugenol and other related compounds against *B. cinerea* [29,30]. The main components of cinnamon essential oil are cinnamaldehyde and *trans*-cinnamaldehyde [27]. Also, different percentages of eugenol can be found depending on the plant parts used for extraction [9,31]. Reports on the efficacy of clove and cinnamon oils against plant pathogens can be found in the literature [27,32,33,34,35]. It was previously found that both oils were effective at suppressing *Rhizoctonia solani* and *Fusarium oxysporum*, and eugenol and cinnamaldehyde are the derivatives, which showed antifungal activity against these pathogens [33]. Cinnamon oil was determined to have moderate antifungal activity against *F. oxysporum* f. sp. *fragariae* [32]. Additionally, a potential application of clove oil in nanoemulsions against *F. oxysporum* f. sp. *lycopersici* was investigated [36]. Cinnamon and clove extracts have the potential to be applied as biofungicides in the future due to their active components, which might be responsible for antifungal activities. As the main components of these two extracts belong to essential oils, the way to extract them should be considered. Supercritical CO_2_ extraction is an advanced and environmentally friendly method to obtain lipophilic compounds from plant material [37]. The possibility to apply clove oil obtained by supercritical CO_2_ extraction in agriculture as a phytotoxic agent was evaluated [26]. Meanwhile, in our study, subcritical CO_2_ extraction was chosen for producing plant extracts. Low process temperature and pressure allow extraction cost to be minimized and active biological compounds to be maintained, thus providing a higher possible fungicidal effect.

The application of the plant extracts as biocontrols on strawberry plants could be a preventative measure to avoid the contamination of fruits, soil and water with chemical residues, as the majority of the extracts are biodegradable and non-toxic. Various alternative ways for the control of *Botrytis cinerea* and other fungal pathogens in greenhouse and field conditions have been investigated [3,38,39,40]. The effectiveness of microbial biocontrol agents and seaweed extract was previously studied on strawberry leaves [41,42,43]. However, in reviewing the available literature, it was observed that there is a lack of studies on the antifungal activity of plant extracts against *B. cinerea* on detached strawberry leaves.

The antifungal activity of cinnamon and clove extracts against *B. cinerea*, especially on detached strawberry leaves, has not been widely investigated. By combining active ingredients from nature with environmentally friendly methods of extraction, new measures for sustainable agriculture could be created. This study aimed to evaluate the antifungal activity of cinnamon (*Cinnamomum cassia* L.) and clove (*Syzygium aromaticum* L.) extracts against strawberry grey mould (*B. cinerea*) in vitro and on detached strawberry leaves and also aimed to identify the chemical composition of volatile compounds.

## 2. Results

The chemical composition of the volatile compounds of CO_2_ extracts of *C. cassia* and *S. aromaticum* is presented in Table 1. In total, 98.86% of the compounds of cinnamon extract were identified and *trans*-cinnamaldehyde was determined to be the dominant compound. Meanwhile, based on 99.81% of the components identified in clove extract, eugenol, eugenol acetate and *trans*-caryophyllene were observed in the highest quantities.

**Figure 1:**
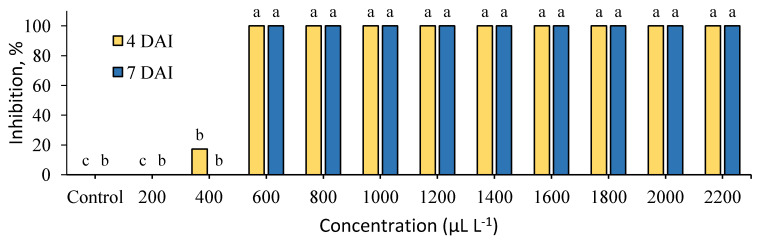
Inhibition (%) of *Botrytis cinerea* mycelial growth by cinnamon (*C. cassia*) extract at 4 and 7 days after inoculation (4 DAI and 7 DAI). Results are presented as means (*n* = 4). The same letter indicates no significant differences between treatments (*p* < 0.05).

The antifungal activity of cinnamon and clove extracts was investigated on PDA (potato dextrose agar) under different concentrations. The inhibition of *B. cinerea* by cinnamon extract is presented in Figure 1. This extract did not demonstrate inhibition at 200 μL L^−1^ at 4 and 7 DAI (days after inoculation) and reached 20% at 400 μL L^−1^ at 4 DAI. However, the mycelial growth of the pathogen was fully inhibited from 600 μL L^−1^ to the full range of concentrations.

**Figure 2:**
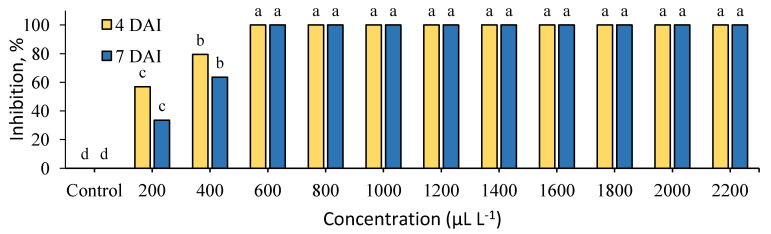
Inhibition (%) of *Botrytis cinerea* mycelial growth by clove (*S. aromaticum*) extract at 4 and 7 days after inoculation (4 DAI and 7 DAI). Results are presented as means (*n* = 4). The same letter indicates no significant differences between treatments (*p* < 0.05).

The inhibition of *B. cinerea* by clove extract is shown in Figure 2. Clove extract expressed stronger antifungal activity at 200 μL L^−1^ and 400 μL L^−1^ and inhibited the growth of *B. cinerea* up to 80% at 4 DAI and 60% at 7 DAI.

Both tested extracts entirely suppressed the mycelial growth at 600–2200 μL L^−1^. The determined MIC (minimal inhibitory concentration) of cinnamon and clove extracts was low and equal to 600 μL L^−1^.

The fungicidal activities of *C. cassia* and *S. aromaticum* extracts were investigated by measuring the radial colony growth of *B. cinerea* after reinoculation. Results are shown in Table 2. Cinnamon extract demonstrated higher fungicidal activity than clove extract. The MFC (minimal fungicidal concentration) of the cinnamon extract was 600 μL L^−1^, as no visible growth of *B. cinerea* was observed 48 h after inoculation, while the MFC of the clove extract was 1400 μL L^−1^. 

**Figure 3:**
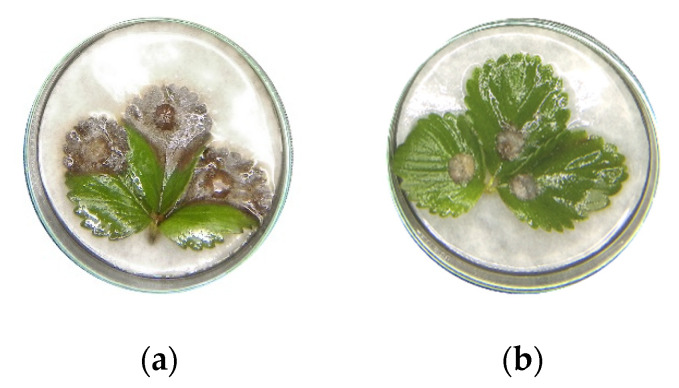
Infection of grey mould on detached strawberry leaves 7 days after inoculation, after treatment with (**a**) cinnamon (*C. cassia*) extract; (**b**) clove (*S. aromaticum*) extract.

**Figure 4:**
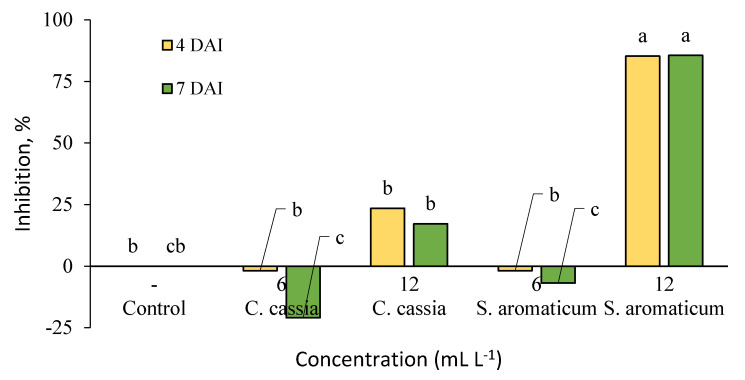
Inhibition (%) of grey mould (*Botrytis cinerea*) by cinnamon (*C. cassia*) and clove (*S. aromaticum*) extracts on detached strawberry leaves at 4 and 7 days after inoculation (4 DAI and 7 DAI). Results are presented as means (*n* = 4). The same letter indicates no significant differences between treatments (*p* < 0.05).

The detached strawberry leaves assay revealed that the clove extract had a higher inhibitory effect of the infection of grey mould (Figure 3). Among all of the investigated treatments, only 12 mL L^−1^ concentration of clove extract significantly reduced the infection on strawberry leaves compared to control (Figure 4). In comparison, 6 mL L^−1^ of both tested extracts increased the spread of grey mould, while 12 mL L^−1^ concentration of the cinnamon extract exhibited a low antifungal effect on detached leaves.

## 3. Discussion

Environmental problems and possible threats to human health resulting from extensive long-term use of chemical fungicides require alternative solutions. Previous research has evaluated the chemical compounds and antifungal activity of essential oils and extracts [32,33,34,35,36,40]. In this research, extracts from cinnamon (*Cinnamomum cassia* L.) and clove (*Syzygium aromaticum* L.) showed high antifungal activity against *Botrytis cinerea* in vitro.

It was determined that *trans*-cinnamaldehyde was the main component in the cinnamon extract, which supports the expected composition of extracts from cinnamon bark [9,35]. Eugenol was a significant compound in the clove bud extract. However, the determined amount of this compound (52.88%) in the volatile fraction of our extract was lower compared to the composition of the essential oils in other studies [25,28]. The differences in the composition may have been a result of the differences in the extraction methods or raw materials; however, the obtained result is opposite to the findings of Yazdani et al. [24], who observed a higher content of eugenol in the clove oil, which was produced by supercritical CO_2_ extraction and then steam distillation. Our clove extract had up to a 10% lower content of eugenol and a slightly higher amount of eugenol acetate compared to the studies of clove essential oils obtained under different supercritical CO_2_ extraction conditions [26].

The unique composition of the extracts provides a possibility for this material to be applied as biopesticides. Other studies [33] confirmed the high antifungal activity of the main constituents of cinnamon essential oil—cinnamaldehyde and clove essential oil—eugenol in vitro. Cinnamon bark oil and cinnamon oil showed strong fumigation and contact activities against the causal agent of false rice smut (*Villosiclava virens*) [35]. Moreover, the main determined component of both oils was *trans*-cinnamaldehyde, which decreased the sporulation of *V. virens*. In our study, *trans*-cinnamaldehyde-rich cinnamon extract showed high antifungal activity against *B. cinerea*. Wang et al. [29] determined that eugenol inhibited the mycelium growth of only *B. cinerea* and Sclerotinia sclerotiorum from ten different fungal plant pathogens. High sensitivity to eugenol containing clove extract could be seen in the results of our study, where high inhibition of *B. cinerea* was observed at the lowest investigated concentrations.

Our results contribute to the findings of Xie et al. [33], whose study showed that clove oil had higher antifungal activity than cinnamon oil against fungal pathogens *Rhizoctonia solani* and *Fusarium oxysporum*. Observed antifungal indexes of cinnamon oil were respectively 54.8% and 28.5% at a 400 μg mL^−1^, while clove oil reached 100.0% and 83.7% at 300 μg mL^−1^. In our study, a broader range of concentrations was investigated, and 100.0% inhibition of *B. cinerea* was achieved at 600 μL mL^−1^. In order to achieve complete inhibition, a higher concentration might be necessary due to the lower concentration of essential oil antifungal components in the whole extract compared to the pure essential oil in Xie et al.’s study [33]. Contrary to our results, Moghadam et al. [27] found cinnamon essential oil (95% cinnamaldehyde) to be more effective against mycotoxin producer *Aspergillus ochraceus* than clove essential oil (93% of eugenol). However, both oils had the highest antifungal activity from all tested oils in the mentioned study. Despite the lower concentration of eugenol in clove extract compared to other studies, high antifungal activity against *B. cinerea* was achieved. The antifungal effect of plant-derived products may vary depending on the pathogen, though our study supports the contribution of the main components of the oils to their effectiveness.

The mechanism of the antifungal activity of cinnamon and clove extract was not identified in this study. Melgarejo-Flores et al. [31] stated that the effectiveness of cinnamon leaf oil (*Cinnamomum zeylanicum*) against *B. cinerea* depends on the ability of the main constituents (eugenol and cinnamaldehyde) to disrupt cell walls and membranes. In this study, cinnamon and clove extract showed low MFC, which describe the ability not only to suppress the growth of the *B. cinerea* temporarily but also to damage it irreversibly. Properties of eugenol from clove oil to break fungal structures are supported by the study of Olea et al. [30]. As the components of both extracts in our study are eugenol and cinnamaldehyde, we could expect the same manner of suppressing/damaging the pathogen. The results of our study agree with the fact that extracts and essential oils rich in eugenol and cinnamaldehyde have high antifungal activities.

The application of seaweed *Ascophyllum nodosum* extract on detached strawberry leaves against spore germination of *Podosphaera aphanis*, the causal agent of powdery mildew, can be found in the literature [43]. To the best of our knowledge, no studies of cinnamon and clove extract antifungal activity have been previously performed on detached strawberry leaves. In the present research, only 12 mL L^−1^ of clove extract managed to inhibit the infection of grey mould on detached strawberry leaves. Cinnamon extract demonstrated low antifungal effect at 12 mL L^−1^; as such, for future experiments, a higher concentration of this extract should be examined for higher effectiveness. For both investigated extracts, increased mycelial growth of *B. cinerea* was observed at 6 mL L^−1^. Environmental stress caused to the pathogen by the extracts may be responsible for this. In that case, the main biological processes of the fungi, including growth, were even more active. We recommend testing higher concentrations of the extracts together with phytotoxicity in the future.

To conclude, clove extract showed the potential to suppress the infection of grey mould on detached strawberry leaves and has the potential to become a biofungicide for the biocontrol of pathogens affecting strawberry. Future research should aim to elucidate optimal concentrations for in situ applications.

## 4. Materials and Methods 

### 4.1. The Extraction of Plant Material

The extracts of cinnamon bark (*Cinnamomum cassia* L.) and clove bud (*Syzygium aromaticum* L.) were chosen for the determination of antifungal activity against strawberry pathogen *Botrytis cinerea* at different concentrations. Dried cinnamon bark and clove bud were obtained from Saldva (Lithuania). Subcritical CO_2_ extraction was performed while producing cinnamon and clove extracts as described in our previous study [44]. Both extracts were kept at 4 °C until the experiments.

### 4.2. Identification of the Extracts Chemical Composition

Volatile compounds of extracts were determined by gas chromatography/mass spectrometry (GC-MS). The analysis was performed on GC-2010Plus/GCMS-QP2010 Ultra system (Shimadzu, Kyoto, Japan) equipped with Rxi-5MS capillary column (30 m × 0.25 mm; film thickness, 0.25 μm) (Restek, Bellefonte, PA, USA). The injector temperature was 250 °C, and the flow rate was 1 mL min^−1^. The column temperature was raised from 50 °C to 160 °C at a rate of 5 °C min^−1^, and then raised until 250 °C at a rate of 10 °C min^−1^. Split mode for samples injection was 1:20. Mass spectra were obtained at 220 °C, in electronic impact mode at 70 eV.

### 4.3. Antifungal Activity In Vitro

Antifungal activity against *B. cinerea* was determined by pouring clove and cinnamon extracts to PDA at concentrations of 200–2200 μL L^−1^. The research was carried out at the Laboratory of Plant Protection, LAMMC Institute of Horticulture, 2018–2019. Four replications were performed for the experiment. Inoculation was performed with a 6-mm plug of 7-day-old single spore isolate of *B. cinerea*, fungal side down. Isolates were obtained from infected strawberry fruit and had previously been identified as *B. cinerea* [45].

Petri dishes were incubated at 22 ± 2 °C in the dark for 7 days. The radial colony growth of the pathogen (plug excluded) was measured 2, 4 and 7 days after inoculation (DAI), and antifungal activity was expressed as the percentage inhibition of mycelial growth using the following formula [46]:

Inhibition (%) = (*C* − *T*)/*C* × 100, (1)

where *C* is the radial colony growth of the pathogen in control (mm); and *T* is the radial colony growth of the pathogen in treatment (mm).

The lowest concentration of cinnamon and clove extract with 100% inhibition was considered as minimal inhibitory concentration (MIC). Minimal fungicidal concentration (MFC) was determined after reinoculating *B. cinerea* treated with extracts from the margin of the colony on the fresh PDA. The lowest concentration with no visible radial colony growth of *B. cinerea* after 48 h was considered as MFC.

### 4.4. Antifungal Activity on Detached Strawberry Leaves

Detached strawberry leaves were soaked in 70% ethanol for 2 minutes and rinsed in sterile distilled water three times. Each leaf was placed in a Petri dish with filter paper and 5 mL of sterile distilled water. The mixtures for leaf treatment were prepared with sterile distilled water, 1% Tween 80 and cinnamon and clove extracts at 6 mL L^−1^ and 12 mL L^−1^. Concentrations were chosen and modified according to the obtained MICs of clove and cinnamon extracts. Detached strawberry leaves were sprayed with prepared mixtures, then wounded with a sterile needle and a 9-mm plug of 7-day-old *B. cinerea* was placed on the wound. Four replications were carried out. Incubation was carried out at 22 ± 2 °C in the dark for 7 days. Evaluations of lesion diameter were made 2, 4 and 7 days after inoculation, and inhibition of grey mould infection on detached strawberry leaves was counted using the formula referred to in Section 4.3, using lesion diameter instead of radial colony growth of the pathogen.

### 4.5. Statistical Analysis

SAS Enterprise Guide 7.1 program (SAS Institute Inc., Cary, NC, USA) was applied for the analysis of experimental data. Analysis of variance (ANOVA) procedure was processed, and Duncan’s multiple range test (*p* < 0.05) was used for the comparison of obtained means.

## 5. Conclusions

Extracts of cinnamon and clove, containing characteristic compounds *trans*-cinnamaldehyde and eugenol, showed significant antifungal activity against *B. cinerea* in vitro. The results showed that the antifungal activity of clove extract was stronger than that of the cinnamon extract at lower concentrations. Infection of grey mould on detached strawberry leaves was suppressed by the application of clove oil at the highest investigated concentration. The cinnamon extract was not that effective at inhibiting the spread of grey mould on strawberry leaves. The use of natural antifungal agents is being increasingly encouraged nowadays. Clove extract has high potential to be applied in the formulation of biopesticides for safer plant protection. Based on the in vitro results, further analysis of cinnamon extract is encouraged.

## Figures and Tables

**Table 1:** Composition of the volatile compounds of cinnamon (*C. cassia*) and clove (*S. aromaticum*) extracts. Results are presented as means (*n* = 3).

Plant Extracts	*Cinnamomum cassia*		*Syzygium aromaticum*	
Component	PA ^1^ (%)	RT ^2^	PA (%)	RT
α-pinene	0.47	6.694	0.26	6.695
Camphene	0.16	7.075		
Benzaldehyde	0.23	7.423		
β-pinene	0.16	7.803		
Limonene	0.15	9.217		
Eucalyptol	0.73	9.279	0.36	9.285
Linalool	0.10	11.261		
Borneol	0.22	13.201		
Terpinen-4-ol	0.18	13.496		
α-terpineol	0.28	13.894		
Chavicol			0.14	15.983
*trans*-cinnamaldehyde	74.67	16.561		
α-cubebene			0.82	18.163
Eugenol	2.55	18.478	52.88	18.787
Cyclosativene	0.19	18.642		
α-copaene	3.51	18.913	0.93	18.935
β-elemene	0.11	19.284		
cis-α-bergamotene	0.85	19.849		
*trans*-caryophyllene	1.13	20.033	17.80	20.168
*trans*-α-bergamotene	0.11	20.353		
Coumarin	6.05	20.659		
α-humulene	0.20	20.887	2.00	20.922
γ-muurolene	0.28	21.418		
α-curcumene	0.10	21.512		
Germacrene D			0.27	21.568
β-selinene	0.11	21.697		
α-muurolene	0.75	21.996		
β-bisabolene	0.10	22.138		
7-epi-α-selinene	0.13	22.456		
*trans*-calamenene + eugenyl acetate + cadinene	1.59	22.584		
Eugenol acetate			21.95	22.822
α-calacorene	0.12	23.043		
Caryophyllene oxide	0.66	24.099	0.52	24.134
Epicubenol	0.22	25.341		
T-muurolol	0.24	25.731		
α-muurolol	0.11	25.828		
α-cadinol	0.10	26.057		
Cadalene	0.11	26.516		
Methyl atrarate	0.13	27.423		
Hexadecenoic acid	0.66	31.061		
Squalene			0.53	33.304
Other ^3^	1.40		1.35	
Total identified	98.86%		99.81%	

^1^ PA—peak area. ^2^ RT—retention time. ^3^ Consists of compounds that were less than 0.1% of the quantity of the extract.

**Table 2:** Radial colony growth of *Botrytis cinerea* 48 h after reinoculation (after treatment with extracts). Results are presented as mean ± SD (*n* = 4) (*p* < 0.05).

Plant Extracts	Radial Colony Growth (mm) at Different Concentrations in μL L^−1^										
	200	400	600	800	1000	1200	1400	1600	1800	2000	2200
*C. cassia*	34.8 ± 1.8	37.0 ± 0.0	0.0 ± 0.0	0.0 ± 0.0	0.0 ± 0.0	0.0 ± 0.0	0.0 ± 0.0	0.0 ± 0.0	0.0 ± 0.0	0.0 ± 0.0	0.0 ± 0.0
*S. aromaticum*	4.0 ± 4.0	43.0 ± 1.5	21.8 ± 4.3	14.3 ± 2.2	15.0 ± 0.5	9.8 ± 1.8	0.0 ± 0.0	0.0 ± 0.0	0.0 ± 0.0	0.0 ± 0.0	0.0 ± 0.0
Control	39.3 ± 0.7										
